# The Effect of Sample Glucose Content on PNGase F-Mediated N-Glycan Release Analyzed by Capillary Electrophoresis

**DOI:** 10.3390/molecules27238192

**Published:** 2022-11-24

**Authors:** Rebeka Torok, Felicia Auer, Robert Farsang, Eszter Jona, Gabor Jarvas, Andras Guttman

**Affiliations:** 1 Translational Glycomics Group, Research Institute of Biomolecular and Chemical Engineering, University of Pannonia, 8200 Veszprem, Hungary; 2 Horváth Csaba Memorial Laboratory of Bioseparation Sciences, Research Center for Molecular Medicine, Faculty of Medicine, Doctoral School of Molecular Medicine, University of Debrecen, 4032 Debrecen, Hungary; 3 Department of Chemistry, Seoul National University, Seoul 03080, Republic of Korea

**Keywords:** capillary gel electrophoresis, PNGase F, N-glycome, immunoglobulin G, Michaelis-Menten kinetics, enzyme activity

## Abstract

Protein therapeutics have recently gained high importance in general health care along with applied clinical research. Therefore, it is important to understand the structure–function relationship of these new generation drugs. Asparagine-bound carbohydrates represent an important critical quality attribute of therapeutic glycoproteins, reportedly impacting the efficacy, immunogenicity, clearance rate, stability, solubility, pharmacokinetics and mode of action of the product. In most instances, these linked N-glycans are analyzed in their unconjugated form after endoglycosidase-mediated release, e.g., PNGase F-mediated liberation. In this paper, first, N-glycan release kinetics were evaluated using our previously reported in-house produced 6His-PNGase F enzyme. The resulting deglycosylation products were quantified by sodium dodecyl sulfate capillary gel electrophoresis to determine the optimal digestion time. Next, the effect of sample glucose content was investigated as a potential endoglycosidase activity modifier. A comparative Michaelis-Menten kinetics study was performed between the 6His-PNGase F and a frequently employed commercial PNGase F product with and without the presence of glucose in the digestion reaction mixture. It was found that 1 mg/mL glucose in the sample activated the 6His-PNGase F enzyme, while did not affect the release efficiency of the commercial PNGase F. Capillary isoelectric focusing revealed subtle charge heterogeneity differences between the two endoglycosidases, manifested by the lack of extra acidic charge variants in the cIEF trace of the 6His-PNGase F enzyme, which might have possibly influenced the glucose-mediated enzyme activity differences.

## 1. Introduction

Protein therapeutics are new-generation drugs produced by the biopharmaceutical industry, utilizing the machinery of various cell lines during bioprocessing [1,2]. These recombinant therapeutic proteins, i.e., the products manufactured with these new technologies, are mostly monoclonal antibodies (mAb) [3]. However, other therapeutic antibody-related products such as Fc-fusion proteins, antibody fragments and antibody–drug conjugates have also become a progressively important part of the biotherapeutics palette [4] and utilized to treat a wide variety of medical conditions including paroxysmal nocturnal hemoglobinuria [5], cryopyrin-associated periodic syndromes [6], different types of cancer [7], multiple sclerosis [8], asthma and rheumatoid arthritis [9], just to mention a few. Their widespread use is reflected in the current market dominance of biopharmaceutical drugs worldwide [10]. Due to the significance of these biological drugs, understanding of their structure–function relationship is of high importance. In the case of glycoprotein therapeutics, one of the important critical quality attributes (CQA) is their N-glycosylation profile [11]. Asparagine-linked carbohydrates have influences on antibody conformation, effector function, stability, solubility, secretion, pharmacokinetics and immunogenicity [12]. The identification and characterization of the attached oligosaccharides are conducted in most instances at the unconjugated glycan level. PNGase F-mediated N-linked carbohydrate release is one of the most extensively used methods in such studies [13,14]. However, it has been reported that the released oligosaccharides and certain sugar ingredients in the sample may influence the endoglycosidase catalyzed reaction, e.g., glucose present in the formulation material [15].

The kinetics of enzyme-mediated biochemical reactions are usually modeled by the Michaelis-Menten method, considering that the enzyme (E) forms an intermediate complex (ES) with the substrate (S), which is later converted to the product (P), while the enzyme is regenerated [16]:

E + S ⇌ ES → E+ P(1)

Therefore, the Michaelis-Menten concept can be readily utilized to study enzymatic reaction kinetics as follows:

(2) $$V=V_{\max}\frac{\left[S\right]}{K_{M}+\left[S\right]}$$

where *V* is the average reaction rate, *V*_max_ is the maximum reaction rate, [S] is the substrate concentration and K_M_ is the Michaelis-Menten constant. This latter (K_M_) allows us to make relative comparisons between the kinetics of different enzymes with the same specificity [17]. In the case of glycoprotein substrates, the reaction products can be rapidly quantified by sodium dodecyl sulfate capillary gel electrophoresis (SDS-CGE), a high-resolution and automated approach to SDS-PAGE that readily separates the deglycosylated and intact proteoforms [18]. As a matter of fact, SDS-CGE is widely used in the biopharmaceutical industry to analyze therapeutic proteins in a simple and robust manner during drug development, production and release testing [19]. Capillary isoelectric focusing (cIEF) is another frequently used bioanalytical method used to gain information about the charge heterogeneity of therapeutic proteins, therefore increasingly adopted in recent years for their characterization [18].

## 2. Results

In this study, first, the digestion reaction kinetics of our in-house produced 6His-PNGase F [20] was evaluated using human immunoglobulin-G1 (hIgG1) as substrate. The digestion products were analyzed by SDS-CGE to follow the reaction speed, and Michaelis-Menten plots were used to shed light on the release efficiency. The Michaelis-Menten constants and the maximum reaction rates were comparatively determined in the presence and absence of glucose in the digestion reaction mixture for a frequently used commercial endoglycosidase and the in-house produced 6His-PNGase F. Finally, capillary isoelectric focusing was employed to study the charge heterogeneity profiles of the two enzymes.

**Figure 1:**
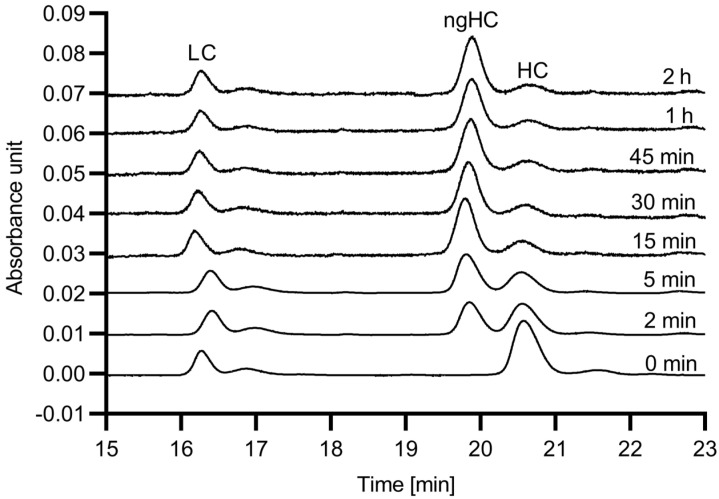
SDS-CGE analysis of the hIgG1 sample with different 6His-PNGase F digestion times at 37 °C. Peaks: LC: light chain, ngHC: non glycosylated heavy chain, HC: heavy chain. Conditions: 30 cm total (20 cm effective) length, 50 µm ID bare fused silica capillary with SDS-MW gel buffer. Applied electric potential: 15 kV (reverse polarity mode); temperature: 25 °C; injection: 5 kV/20 s.

First, the digestion efficiency of the 6His-PNGase F enzyme was evaluated with respect to reaction speed using hIgG1 as substrate. The digestion reactions were conducted up to 120 min, and SDS-CGE was used to quantify the release efficiency, based on the resulting peak area changes for the glycosylated and non-glycosylated heavy chain fragments. Figure 1 shows the obtained electropherograms.

**Figure 2:**
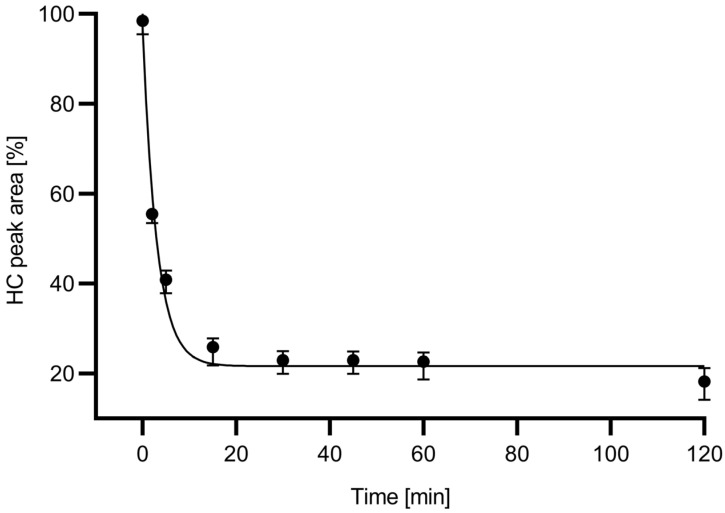
Deglycosylation time plot showing the peak area decrease of the heavy chain fragment during the endoglycosidase reaction using the in-house produced 6His-PNGase F. All measurements were made in triplicates.

As the endoglycosidase digestion reaction proceeded, the peak area of the glycosylated mAb heavy chain fragment (HC) decreased, while the peak representing the non-glycosylated heavy chain (ngHC) increased. Please note that almost full deglycosylation occurred within the first 15 min. Figure 2 shows the digestion time plot based on the peak area decrease in the HC subunit with the use of the in-house produced 6His-PNGase F enzyme.

Based the deglycosylation time plot in Figure 2, a 15 min digestion time was chosen for all downstream experiments because during this time frame, apparently close to maximum release rate was already achieved. The relative ngHC peak area % was calculated as depicted by Equation (3) and used accordingly in all further calculations.

(3) $$\mathrm{relative}\;\mathrm{ngHC}\;\mathrm{peak}\;\mathrm{area}\;\%=\frac{\mathrm{ngHC}\;\mathrm{peak}\;\mathrm{area}\times 100}{\mathrm{ngHC}\;\mathrm{peak}\;\mathrm{area}+\mathrm{HC}\;\mathrm{peak}\;\mathrm{area}}$$

**Figure 3:**
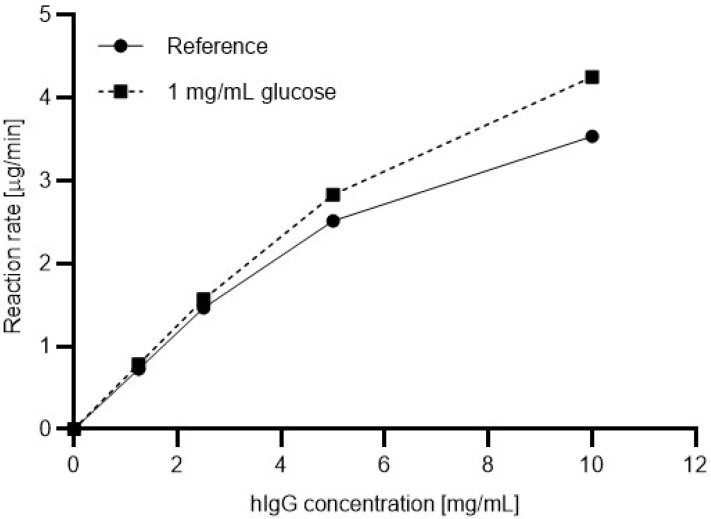
Michaelis-Menten deglycosylation plots using the in-house produced 6His-PNGase F enzyme with (dashed line) and without (solid line, reference) the presence of glucose (1 mg/mL) in the reaction mixture.

Next, the Michaelis-Menten plots were generated with the presence and absence of glucose in the reaction mixtures. Four different substrate concentrations were prepared ranging from 1.5 mg/mL to 10 mg/mL. Please note that the use of a substrate concentration higher than 10 mg/mL resulted in precipitation during the denaturation step. To understand the effect of the sugar content in the sample on the reaction kinetics, glucose was added to the reaction mixtures in 1 mg/mL final concentration. Figure 3 shows the resulting Michaelis-Menten plots based on the relative ngHC peak area % values (Equation (3)), without (solid line) and with (dashed line) the presence of 1 mg/mL glucose in the digestion reaction using the in-house produced 6His-PNGase F. The reaction rates were determined as follows:

(4) $$\mathrm{Reaction}\;\mathrm{rate}=\frac{\mathrm{hIgG}1\;\mathrm{concentration}\cdot \mathrm{relative}\;\mathrm{ngHC}\;\mathrm{area}}{\mathrm{digestion}\;\mathrm{time}}$$

As one can observe, with increasing substrate concentration, the reaction rate increased in a higher degree with the presence of glucose in the reaction mixture. Apparently, with 10 mg/mL hIgG1 substrate concentration, the addition of 1 mg/mL glucose resulted in a 16.8% improvement in the reaction rate. Therefore, glucose should be considered as an activator for the 6His-PNGase F enzyme.

**Figure 4:**
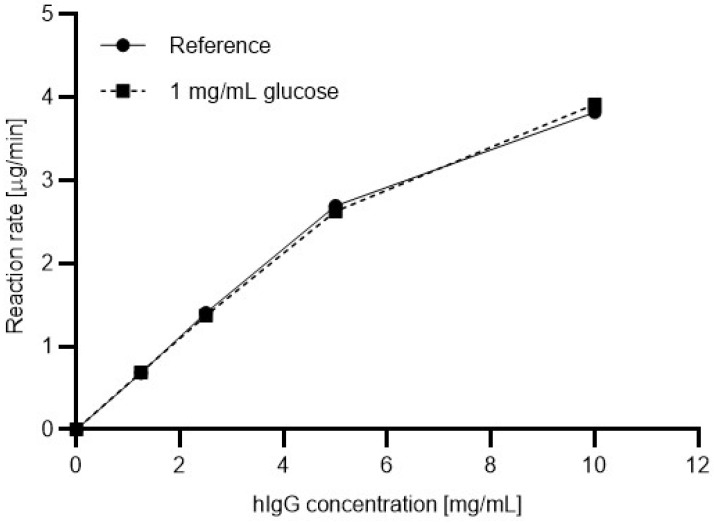
Michaelis-Menten deglycosylation plots using a commercial PNGase F enzyme with (dashed line) and without (solid line, reference) the addition of glucose (1 mg/mL) to the reaction mixture.

Based on the results shown in Figure 3, the digestion efficiency was also evaluated for a commonly used commercial PNGase F enzyme. Figure 4 shows the resulting Michaelis-Menten plots for the commercial enzyme with (dashed line) and without (solid line, reference) 1 mg/mL glucose in the reaction mixture. In this instance, apparently no significant differences were found between the reaction rates.

For comparative purposes, the maximum reaction rates (*V*_max_) and the Michaelis-Menten constants (K_M_) were calculated from Figure 3 and Figure 4 for both enzymes with and without the presence of 1 mg/mL glucose in the reaction mixture using the GraphPad Prism software (GraphPad Software, San Diego, CA, USA). Please note that these values were predicted by the software since the precipitation of the IgG at a concentration higher than 10 mg/mL hampered further measurements. The significantly increasing reaction rate (activation) with the in-house produced 6His-PNGase F (UP) in the presence of 1 mg/mL glucose (Figure 3) was in contrast to the less than moderate change with the use of the commercial enzyme (Figure 4), as the corresponding maximum reaction rates and Michaelis-Menten constants depict in Table 1.

As shown by the data in Table 1, the *V*_max_ value of the commercial enzyme was 23% higher than that of the 6His-PNGase F without having glucose in the reaction mixture. On the other hand, when 1 mg/mL glucose was present in the digestion reaction, the 6His-PNGase F performed slightly (3%) better than its commercial counterpart with the application of the same enzyme units. More interestingly, the addition of glucose increased the reaction rate of the commercial enzyme only by 12% but showed significant activation (43%) in the performance of the 6His-PNGase F. The associated K_M_ values followed the same trend according to the Michaelis-Menten theory.

**Figure 5:**
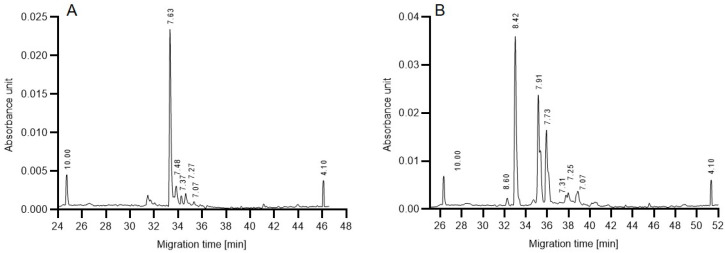
Capillary isoelectric focusing analysis of the in-house produced 6His-tagged (**A**) and a commercial (**B**) PNGase F enzymes. Numbers above the peaks represent the corresponding pI values. Separation conditions: 20 cm effective capillary length (30 cm total length, 50 µM ID, NHCO coating); applied focusing and mobilization electric field strengths were 25 kV and 30 kV, respectively. pI markers: 4.1 and 10.0. Separation temperature: 20 °C. The sample was mixed into the Master Mixture and introduced by applying 25 psi for 99 s, i.e., to completely fill the separation capillary.

As a first step towards gaining some preliminary understanding of the above described reaction rate differences, isoelectric focusing (cIEF) was performed to compare the charge heterogeneities of the in-house produced 6His-PNGase F and the commercial enzyme. The resulting separation traces in Figure 5 reveal subtle differences between the profiles. Although the functional group of both endoglycosidases is assumed to be similar, the in-house produced 6His-PNGase F enzyme (Figure 5A) featured only one dominant peak at pH 7.63 with no significant acidic and basic variants. Please note that the His-tag is known to shift the pI of the conjugate towards the acidic region. In the cIEF trace of the commercial enzyme (Figure 5B), on the other hand, two major acidic isoforms appeared (pHs 7.91 and 7.73) in addition to the main peak at pH 8.42. As a first approximation, we consider that the presence and absence of these acidic variants may be somewhat responsible for the observed activity variations in the presence of glucose during the deglycosylation reaction.

## 3. Conclusions

In this work, the deglycosylation kinetics and the effect of sample glucose content were studied on the enzymatic N-linked carbohydrate release reaction comparing the in-house produced 6His-tagged and a commercial PNGaseF enzyme. The deglycosylation rate of the human IgG1 heavy chain substrate was quantitatively followed by SDS-CGE. The performance of the two endoglycosidases were tested without and with the presence of 1 mg/mL glucose in the reaction mixtures with increasing substrate (hIgG1) concentrations. The comparative Michaelis-Menten plots were evaluated for both. While the commercial enzyme did not exhibit a significant glucose-dependent reaction rate change, the in-house produced 6HIS-tagged enzyme performed >40% better in the presence of glucose, which therefore, can be considered as an activator. Although the functional regions of the two enzymes were assumable similar, the differences in their glucose-dependent enzyme activity may have been due to the structural disparity that was initially analyzed by cIEF in this study. The charge heterogeneity pattern of the commercial enzyme was more complex with two additional acidic variants in addition to the main component in comparison to the in-house produced 6His-tagged PNGase F that featured only one major peak.

## 4. Materials and Methods

### 4.1. Chemicals and Reagents

Sodium hydroxide, phosphoric acid, glacial acetic acid, urea, iminodiacetic acid, 2-mercaptoethanol and L-arginine were from Sigma Aldrich (St. Louis, MO, USA). The Pharmalyte 3–10 was from GE Healthcare (Chicago, IL, USA). The cIEF Gel; the pI markers of the pI Peptide Marker kit; the Fast Glycan kit; and the SDS-MW Analysis Assay kit including the SDS-MW Gel Buffer, Sample Buffer and 10 kDa protein standard along with the 50 µm ID NCHO and BFS capillaries were from Bio-Science Kft (Budapest, Hungary). The hIgG1 test sample was from Molecular Innovations (Novi, MI, USA). The commercial PNGase F enzyme was from New England Biolabs (Ipswich, MA, USA). The in-house produced 6His-PNGase F (production described in detail in [20] and this enzyme featured 3 months of shelf life) was from University of Pannonia (Veszprem, Hungary).

### 4.2. PNGase F Digestion

During the enzyme kinetic study, 1.5–10 µL of 10 mg/mL hIgG1 was used as the substrate. First, the sample was diluted to 10 µL (if necessary) and denatured by the addition of 2.0 µL of a denaturation mixture (Fast Glycan Kit, Bio-Science Kft, Budapest, Hungary) followed by incubation at 70 °C for 15 min. Then, 20 µL of 18 mM ammonium acetate was added to the denatured samples, and the N-glycans were released by the addition of 1 µL of the 6His-tagged or the commercial PNGase F enzyme (1.5 mU). The reaction mixture was incubated at 37 °C for the time periods shown in the corresponding parts of the manuscript. The endoglycosidase digestion reaction was stopped by the addition of the SDS-MW denaturation buffer.

### 4.3. Sample Preparation for SDS-CGE and cIEF

For reduced protein sample preparation, 80 µL of SDS-MW Sample Buffer was mixed with 5.0 µL of 2-mercaptoethanol, 2.0 µL of 10 kDa marker and 10 µL of the undigested or PNGase F digested (each individual time points) glycoprotein solutions followed by incubation at 70 °C for 15 min in a PCR instrument with heated lid.

The cIEF Master Mix contained the cIEF gel, 3.75 M urea, 48 mL of Pharmalyte 3–10, 80 mL of 500 mM L-arginine, 8.0 mL of 200 mM iminodiacetic acid, 1-1 mL of pI Marker 4.1 and 10.0 each; 100 µL of Master Mix and 2.0 µL of 10 mg/mL sample protein were mixed prior to the cIEF measurements.

### 4.4. SDS-CGE Analysis

An MDQ Capillary Electrophoresis System (Beckman Coulter, Fullerton, CA, USA) was used with UV detection (220 nm) for all SDS-CGE analyses, employing the SDS-MW Gel Buffer in a 20 cm effective length (30 cm total length), 50 μm ID bare fused capillary. The column was conditioned prior to each measurement and between runs by rinsing for 5 min each with HPLC grade water, 0.5 M NaOH and 0.5 M HCl. The separation voltage was set to 15 kV in reverse polarity mode (cathode at the injection side). The samples were injected by applying 5.0 kV for 20 s, also in reverse polarity mode. The separation temperature was 25 °C. For data acquisition and analysis, the 32Karat (version 8.0) software package (Beckman Coulter, Fullerton, CA, USA) was used. The GraphPad Prism (8.0.1 version, GraphPad Software, San Diego, CA, USA) software was used for the calculation of *V*_max_ and K_M_ values.

### 4.5. cIEF Analysis

The same capillary electrophoresis system was used for all cIEF measurements but with UV detection at 280 nm. For the separations, a 50 µM ID, 30 cm total length (20 cm effective length) NHCO capillary was employed. The sample storage compartment was set to 10 °C. Prior to injections; the capillary was washed with 6 M urea at 50 psi for 5.0 min followed by HPLC grade water at 50 psi for 5.0 min. The injection was achieved by filling up the capillary with the sample, mixed into the Master Mixture (25 psi/100 s). The formation of the pH gradient and focusing started by applying an 833 V/cm electric field in normal polarity mode for 15 min between the anolyte (200 mM phosphoric acid) and catholyte (300 mM sodium hydroxide) solutions. Then, the mobilization was performed using 1000 V/cm electric field (30 kV) in normal polarity mode between the anolyte and the chemical mobilizer (350 mM acetic acid) for 45 min at 20 °C capillary cartridge temperature. Data acquisition and analysis were completed as described above.

## Figures and Tables

**Table 1:** Maximum reaction rates (*V*_max_) and Michaelis-Menten constants (K_M_) based on the results in Figure 3 and Figure 4 for the in-house produced 6His-PNGase F and the commercial PNGase F enzymes, respectively. The “reference” columns represent the values without 1 mg/mL glucose in the reaction mixture.

	Commercial PNGase F		6His-PNGase F	
	Reference	With Glucose	Reference	With Glucose
*V*_max_ [µg/min]	8.39 ± 0.17	9.43 ± 0.12	6.80 ± 0.07	9.73 ± 0.17
K_M_ [nM]	81 ± 2.2	90 ± 2.5	59 ± 1.7	85 ± 2.3

## Data Availability

The data are available upon request from the authors.
